# Association Between Bedtime at Night and Systolic Blood Pressure in Adults in NHANES

**DOI:** 10.3389/fmed.2021.734791

**Published:** 2021-12-24

**Authors:** Yingjie Su, Changluo Li, Yong Long, Liudang He, Ning Ding

**Affiliations:** Department of Emergency Medicine, The Affiliated Changsha Central Hospital, Hengyang Medical School, University of South China, Changsha, China

**Keywords:** bedtime, systolic blood pressure, hypertension, gender, NHANES

## Abstract

**Objectives:** This study aimed to explore the association between bedtime at night and systolic blood pressure (SBP) in adults.

**Methods:** We conducted a cross-sectional study composed of 7,642 individuals from the National Health and Nutrition Examination Survey (NHANES). Bedtime was defined as the response to the question: “What time do you usually fall asleep on weekdays or workdays?” SBP was taken using the average of all measured values. Multiple linear regression analyses were done to explore the relationship between bedtime and SBP.

**Results:** The bedtime was changed from categorical variable to continuous variable for data analysis, and a significantly negative association was identified between bedtime and SBP (β, −0.23 [95% CI, −0.43, −0.02]). With the delay of bedtime, the SBP showed a gradual decrease trend, and it was dropped to the lowest at 0:00. After 0:00, the SBP was gradually increased with the delay of sleep time. The stratified analyses showed that in the female group, with the delay of bedtime, the range of SBP was decreased more obviously at 0:00. In the 18–45 year group, bedtime had little effect on SBP. Among ≥45 years old group, this trend was still the same. In the black group, an obvious downward trend was found at 22:00.

**Conclusion:** With the delay of bedtime, the SBP had shown a gradual decrease trend, and it was dropped to the lowest at 0:00. After 0:00, the SBP was gradually increased with the delay of sleep time. Bedtime and SBP showed a U-shaped relationship.

## Introduction

Hypertension has been a global health problem, which is caused by a combination of genetic and environmental factors (1). In the United States, nearly one-third of adults have suffered from hypertension (2). In recent years, the prevention and treatment of hypertension through lifestyle changes have evoked the awareness of people. Sleep has been identified as a key lifestyle risk factor for cardiovascular disease (3). Sleep can be evaluated from multiple perspectives, including duration, quality, regularity, time, and the existence of sleep disorders. Since sleep duration is the simplest and most direct measure of sleep, it has been utilized as a goal to explore the relationship between sleep and health (4). Related research studies have clarified that the proportion of American adults who sleep less than 7–8 h a day had increased from 20–23% in 1985 to nearly 30% in 2005–2007 (5, 6). Many studies also have shown that blood pressure (BP) was affected by sleep duration (7–11).

The 24 h sleep-wake cycle was closely related to BP and was also affected by several endogenous circadian rhythms and circulatory exogenous factors, which was a coordinated process involving rhythmic changes in sensory, motor, autonomy, endocrine, and brain processes. This change had a certain impact on cardiovascular function and BP (12). Moreover, there were obvious circadian rhythms in BP fluctuations, which were increased during the day and decreased in the middle of the night. Falling asleep too early or too late could disrupt this pattern, and the lack of this normal decline pattern was believed to be a better predictor of cardiometabolic disease risk and mortality (13, 14).

However, few studies have explored the effect of bedtime on BP. Hence, this study aimed to explore the relationship between bedtime at night and systolic BP (SBP).

## Methods

### Study Population

**Figure 1:**
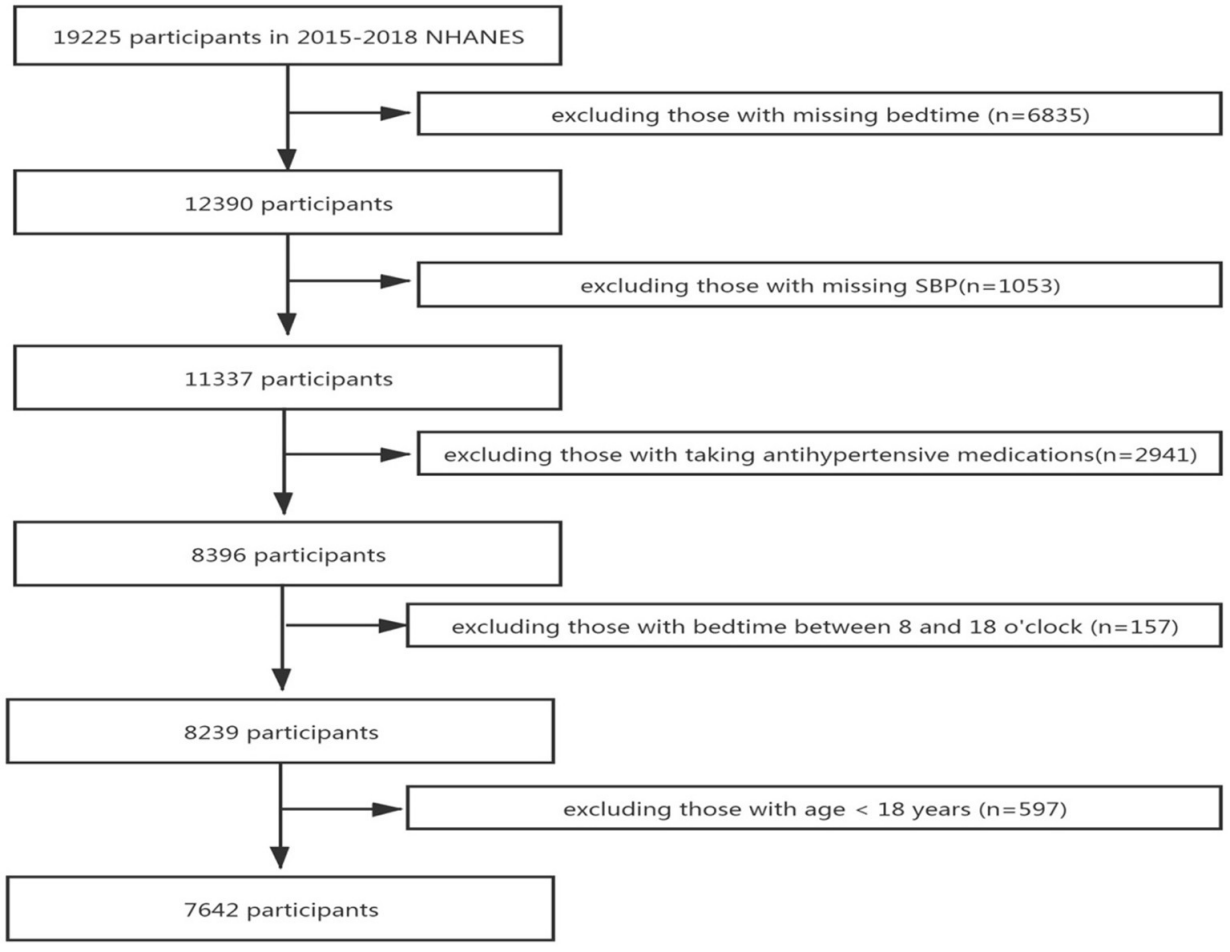
Flowchart of the study design and participants excluded from the study.

We retrieved two cycles of data from 2015 to 2018 in the National Health and Nutrition Examination Survey (NHANES) database for research. A total of 19,225 potential participants were included in the study, and 11,583 participant were excluded for the following reasons: missing bedtime (*n* = 6,835), missing SBP data (*n* = 1,053), taking antihypertensive medications (*n* = 2,941), bedtime between 8:00 and 18:00 o'clock (*n* = 157), and age less than 18 years (*n* = 597). Finally, 7,642 participants were included in the study, after all exclusion criteria were met (Figure 1).

### Definition

Bedtime was defined as the response to the question: “What time do you usually fall asleep on weekdays or workdays?”, the responses were categorized into 13 groups:19:00, 20:00, 21:00, 22:00, 23:00, 0:00, 1:00, 2:00, 3:00, 4:00, 5:00, 6:00, and 7:00. Moreover, it was changed into a continuous variable for data analysis.

Well-trained and certified inspectors used standardized protocols and calibrated equipment to obtain BP readings. Three consecutive BP readings were obtained through auscultation. If the BP measurement was not completed successfully, the fourth measurement would be performed. The SBP was the average of all available measurement data.

### Covariates

In our research, sociodemographic, lifestyle, metabolic, and sleep factors were collected. Sociodemographic factors included race, age, and gender. The race was classified into 4 groups: Mexican American, White, Black, and other race. Age was classified into 3 groups: young (18–44), middle-aged (45–59), and old (≥60). Lifestyle factors included alcohol consumption, smoke, and total physical activity (TPA). Alcohol consumption was defined as the response to the question: “In the past 12 months, how often did you drink any type of alcoholic beverage?”, and the responses were divided into 3 groups: drinking, no drinking, and not recorded. Smoking was defined as the response to the question: “Do you now smoke cigarettes?”, and the responses were divided into 3 groups: smoking, no smoking, and not recorded. According to the physical activity guideline and previous literature (15, 16), the TPA was divided into 5 groups: <150, 150–300, 300–450, ≥450 min, and not recorded. Metabolic factors included glomerular filtration rate (GFR), urea acid (UA), total cholesterol (TC), low-density lipoprotein (LDL), high-density lipoprotein (HDL), body mass index (BMI), and diabetes. GFR was estimated by the simplified Modification of Diet in Renal Disease (MDRD) equation: 186 × SC^−1.154^ × Age^−0.203^× (0.742 if female) (17). According to the WHO guidelines, BMI was divided into underweight (<18.5 kg/m^2^), normal weight (18.5–24.99 kg/m^2^), overweight (25–29.99 kg/m^2^), and obesity (≥30 kg/m^2^) (18). Diabetes was defined as the responses to the question: “Have you ever been told by a doctor or health professional that you have diabetes or sugar diabetes?”, and the responses were divided into 4 groups: yes, no, borderline, and not recorded. The method of obtaining other covariates, including UA, TC, LDL, HDL, can be found at www.cdc.gov/nchs/nhanes/.

### Sleep Factors

Sleep duration was defined as the responses to the question: “How much sleep do you usually get at night on weekdays or workdays?”, and based on the guideline (19), the responses were categorized into 3 groups: <7 h, ≥7 h, and not recorded. Snore was based on the responses to the question: “In the past 12 months, how often did you snore while you were sleeping?” Trouble sleeping was based on the responses: “Have you ever told a doctor or other health professional that you have trouble sleeping?” Snort or stop breathing was based on the responses to the question: “in the past 12 months, how often did you snort, gasp, or stop breathing while you were asleep?” Overly sleepy was defined as the responses to the question: “In the past month, how often did you feel excessively or overly sleepy during the day?” The above 4 variables, namely, snore, trouble sleeping, snort or stop breathing, and overly sleepy, were divided into 3 groups according to the answers: yes, no, and not recorded.

### Statistical Analysis

A multivariate linear regression model was used to assess the correlation between bedtime at night and SBP in adults. The covariates mentioned above were adjusted as potential confounders. The continuous variables were represented by mean ± SD (normal distribution) or median (quartile) (skew distribution), which included GFR, UA, TC, LDL, HDL, and SBP. Categorical variables were represented by percentage or frequency, which included age, race, alcohol consumption, smoke, TPA, BMI, diabetes, sleep duration, snore, trouble sleeping, snort or stop breathing, overly sleepy, and bedtime. To calculate the differences between male and female, a linear regression model was used for continuous variables or chi-square tests for categorical variables. The values of missing continuous covariates were indicated by dummy variables, including GFR, UA, TC, LDL, HDL, and the missing ratios were 6.4, 6.4, 6.2, 57.4, and 6.2%, respectively. The missing categorical variables were included in the analysis as a single group, including alcohol consumption, smoking, TPA, BMI, diabetes, sleep duration, snore, trouble sleeping, snort or stop breathing, and being overly sleepy. We changed the bedtime from categorical variable to continuous variable for data analysis. Stratified analysis and smooth curve fittings were conducted to explore the relationship between bedtime and SBP based on gender, age, race, and BMI. The statistical software packages R (http://www.R-project.org) and EmpowerStats (http://www.empowerstats.com) were used for the data analyses. When the *P*-value was < 0.05, it was considered statistically significant.

## Results

### Participants Characteristics

Table 1 shows the description of sociodemographic and baseline characteristics. Our study included 7,642 participants subclassified based on gender. Among the participants, the proportion of male and female was 48.64% (*n* = 3,717) and 51.36% (*n* = 3,925), respectively. In ethnicity, the proportion of Mexican American, White, and Black was 17.43, 32.92, and 19.34%, respectively. Overall, the mean (SD) values for GFR, UA, TC, LDL, HDL, and SBP were 100.35 (27.49) ml/min/1.73 m^2^, 313.49 (82.47) μmol/L, 4.90 (1.06) mmol/L, 2.91 (0.90) mmol/L, 1.40 (0.42) mmol/L, and 121.59 (17.21) mmHg, respectively. In the participants, 65.18% were alcohol drinkers, 18.61% were smokers, 55.51% were young, 30.31% were normal weight, and 9.83% were diabetes (yes/borderline). Table 2 shows the description of sleep factors. 22.09% of participants were sleep duration with <7 h, 65.60% were snored, 22.18% have trouble sleeping, 21.08% have snorted or stopped breathing, and 77.81% were overly sleepy. Table 3 shows the results of the univariate analysis of SBP.

**Table 1:** Description of 7,642 participants included in the present study.

	**Total (*n* = 7,642)**	**Male (*n* = 3,717)**	**Female (*n* = 3,925)**	***P-value***
**Sociodemographic factors**				
Age (years)				<0.001
18–44	4,242 (55.51%)	1,993 (53.62%)	2,249 (57.30%)	
45–59	1,737 (22.73%)	830 (22.33%)	907 (23.11%)	
≥ 60	1,663 (21.76%)	894 (24.05%)	769 (19.59%)	
Race				0.222
Mexican american	1,332 (17.43%)	636 (17.11%)	696 (17.73%)	
White	2,516 (32.92%)	1,266 (34.06%)	1,250 (31.85%)	
Black	1,478 (19.34%)	713 (19.18%)	765 (19.49%)	
Other race	2,316 (30.31%)	1,102 (29.65%)	1,214 (30.93%)	
**Lifestyle factors**				
Alcohol consumption				<0.001
No drinking	1,039 (13.60%)	544 (14.64%)	495 (12.61%)	
Drinking	4,981 (65.18%)	2,579 (69.38%)	2,402 (61.20%)	
Not recorded	1,622 (21.22%)	594 (15.98%)	1,028 (26.19%)	
Smoke				<0.001
Smoking	1,422 (18.61%)	859 (23.11%)	563 (14.34%)	
No smoking	1,465 (19.17%)	914 (24.59%)	551 (14.04%)	
Not recorded	4,755 (62.22%)	1,944 (52.30%)	2,811 (71.62%)	
TPA (minutes)				<0.001
<150	1,212 (15.86%)	465 (12.51%)	747 (19.03%)	
≥150, <300	340 (4.45%)	135 (3.63%)	205 (5.22%)	
≥300, <450	289 (3.78%)	134 (3.61%)	155 (3.95%)	
≥450	1,779 (23.28%)	1,015 (27.31%)	764 (19.46%)	
Not recorded	4,022 (52.63%)	1,968 (52.95%)	2,054 (52.33%)	
**Metabolic factors**				
GFR (ml/min/1.73m^2^)	100.35 ± 27.49	97.29 ± 25.07	103.24 ± 29.31	<0.001
UA (umol/L)	313.49 ± 82.47	356.13 ± 75.89	273.19 ± 66.67	<0.001
TC (mmol/L)	4.90 ± 1.06	4.88 ± 1.08	4.92 ± 1.04	0.080
LDL (mmol/L)	2.91 ± 0.90	2.95 ± 0.91	2.88 ± 0.90	0.013
HDL (mmol/L)	1.40 ± 0.42	1.26 ± 0.37	1.52 ± 0.43	<0.001
SBP (mmHg)	121.59 ± 17.21	124.14 ± 16.32	119.18 ± 17.68	<0.001
BMI (kg/m^2^)				<0.001
<18.5	162 (2.12%)	64 (1.72%)	98 (2.50%)	
18.5–24.9	2,316 (30.31%)	1,091 (29.35%)	1,225 (31.21%)	
25–29.9	2,375 (31.08%)	1,309 (35.22%)	1,066 (27.16%)	
≥30	2,714 (35.51%)	1,210 (32.55%)	1,504 (38.32%)	
Not recorded	75 (0.98%)	43 (1.16%)	32 (0.82%)	
Diabetes				0.026
Yes/Borderline	751 (9.83%)	395 (10.63%)	356 (9.07%)	
No	6,886 (90.11%)	3,321 (89.35%)	3,565 (90.83%)	
Not recorded	5 (0.07%)	1 (0.03%)	4 (0.10%)	

*GFR, glomerular filtration rate; UA, uric acid; SBP, systolic blood pressure; BMI, body mass index; HDL, high-density lipoprotein; TC, total cholesterol; LDL, low-density lipoprotein; TPA, total physical activity. Mean +/- SD for: GFR, UA, SBP, TC, LDL, HDL. P-value was calculated by linear regression model. % for: race, alcohol consumption, smoke, age, BMI, diabetes, TPA. P-value was calculated by chi-square test*.

**Table 2:** Sleep factors description of 7,642 participants included in the present study.

	**Total (*n* = 7,642)**	**Male (*n* = 3,717)**	**Female (*n* = 3,925)**	***P-value***
**Sleep factors**				
Sleep duration (hours)				<0.001
<7	1,688 (22.09%)	934 (25.13%)	754 (19.21%)	
≥ 7	5,946 (77.81%)	2,778 (74.74%)	3,168 (80.71%)	
Not recorded	8 (0.10%)	5 (0.13%)	3 (0.08%)	
Snore				<0.001
No	2,138 (27.98%)	839 (22.57%)	1,299 (33.10%)	
Yes	5,013 (65.60%)	2,644 (71.13%)	2,369 (60.36%)	
Not recorded	491 (6.43%)	234 (6.30%)	257 (6.55%)	
Trouble sleeping				<0.001
Yes	1,695 (22.18%)	723 (19.45%)	972 (24.76%)	
No	5,943 (77.77%)	2,993 (80.52%)	2,950 (75.16%)	
Not recorded	4 (0.05%)	1 (0.03%)	3 (0.08%)	
Snort or stop breathing				<0.001
No	5,678 (74.30%)	2,593 (69.76%)	3,085 (78.60%)	
Yes	1,611 (21.08%)	948 (25.50%)	663 (16.89%)	
Not recorded	353 (4.62%)	176 (4.74%)	177 (4.51%)	
Overly sleepy				<0.001
No	1,365 (17.86%)	739 (19.88%)	626 (15.95%)	
Yes	5,946 (77.81%)	2,975 (80.04%)	3,296 (83.97%)	
Not recorded	8 (0.10%)	3 (0.08%)	3 (0.08%)	
Bedtime				<0.001
19 pm	56 (0.7%)	33 (0.9%)	23 (0.6%)	
20 pm	256 (3.3%)	128 (3.4%)	128 (3.3%)	
21 pm	1,063 (13.91%)	505 (13.59%)	558 (14.22%)	
22 pm	2,195 (28.72%)	986 (26.53%)	1,209 (30.80%)	
23 pm	1,941 (25.40%)	928 (24.97%)	1,013 (25.81%)	
0 am	1,136 (14.87%)	569 (15.31%)	567 (14.45%)	
1 am	431 (5.64%)	228 (6.13%)	203 (5.17%)	
2 am	267 (3.5%)	150 (4.0%)	117 (3.0%)	
3 am	131 (1.7%)	80 (2.2%)	51 (1.3%)	
4 am	64 (0.8%)	40 (1.1%)	24 (0.6%)	
5 am	30 (0.4%)	23 (0.6%)	7 (0.2%)	
6 am	30 (0.4%)	21 (0.6%)	9 (0.2%)	
7 am	42 (0.5%)	26 (0.7%)	16 (0.4%)	

*% for: sleep duration, snore, trouble sleeping, snort or stop breathing, overly sleepy, bedtime. P-value was calculated by chi-square test*.

**Table 3:** Univariate analysis for systolic blood pressure.

	**Statistics**	**β, (95%CI), P**
Gender		
Male	3,717 (48.64%)	Ref
Female	3,925 (51.36%)	−4.96 (−5.73, −4.20) <0.0001
Race		
White	2,516 (32.92%)	Ref
Mexican american	1,332 (17.43%)	−0.61 (−1.75, 0.53) 0.2927
Black	1,478 (19.34%)	3.79 (2.69, 4.89) <0.0001
Other race	2,316 (30.31%)	−1.55 (−2.51, −0.58) 0.0017
Age (years)		
18–44	4,242 (55.51%)	Ref
45–59	1,737 (22.73%)	9.39 (8.52, 10.27) <0.0001
≥60	1,663 (21.76%)	17.30 (16.41, 18.19) <0.0001
Alcohol consumption		
No drinking	1,039 (13.60%)	Ref
Drinking	4,981 (65.18%)	−4.62 (−5.77, −3.47) <0.0001
Not recorded	1,622 (21.22%)	−5.08 (−6.41, −3.75) <0.0001
TPA (minutes)		
<150	1,212 (15.86%)	Ref
≥150, <300	340 (4.45%)	−2.00 (−4.07, 0.06) 0.0572
≥300, <450	289 (3.78%)	−2.97 (−5.17, −0.77) 0.0082
≥450	1,779 (23.28%)	−4.43 (−5.68, −3.18) <0.0001
Not recorded	4,022 (52.63%)	−3.51 (−4.61, −2.40) <0.0001
Smoke		
Smoking	1,422 (18.61%)	Ref
No smoking	1,465 (19.17%)	2.62 (1.38, 3.87) <0.0001
Not recorded	4,755 (62.22%)	−3.05 (−4.06, −2.04) <0.0001
BMI (kg/m^2^)		
18.5–24.9	2,316 (30.31%)	Ref
<18.5	162 (2.12%)	−3.84 (−6.54, −1.14) 0.0053
25–29.9	2,375 (31.08%)	3.72 (2.75, 4.69) <0.0001
≥30	2,714 (35.51%)	6.58 (5.64, 7.52) <0.0001
Not recorded	75 (0.98%)	11.92 (8.02, 15.81) <0.0001
Diabetes		
No	6,886 (90.11%)	Ref
Yes/Borderline	751 (9.83%)	6.80 (5.51, 8.09) <0.0001
Not recorded	5 (0.07%)	−14.80 (−29.78, 0.18) 0.0529
GFR (ml/min/1.73m^2^)	100.35 ± 27.49	−0.13 (−0.15, −0.12) <0.0001
UA (umol/L)	313.49 ± 82.47	0.04 (0.04, 0.04) <0.0001
TC (mmol/L)	4.90 ± 1.06	2.87 (2.51, 3.24) <0.0001
LDL (mmol/L)	2.91 ± 0.90	3.08 (2.43, 3.73) <0.0001
HDL (mmol/L)	1.40 ± 0.42	−0.94 (−1.87, −0.01) 0.0478
Sleep duration (hours)		
<7	1,688 (22.09%)	Ref
≥7	5,946 (77.81%)	−2.22 (−3.15, −1.29) <0.0001
Not recorded	8 (0.10%)	−1.74 (−13.67, 10.20) 0.7756
Snore		
No	2,138 (27.98%)	Ref
Yes	5,013 (65.60%)	4.38 (3.52, 5.25) <0.0001
Not recorded	491 (6.43%)	6.24 (4.56, 7.91) <0.0001
Trouble sleeping		
Yes	1,695 (22.18%)	Ref
No	5,943 (77.77%)	−0.86 (−1.79, 0.07) 0.0707
Not recorded	4 (0.05%)	0.08 (−16.81, 16.96) 0.9930
Snort or stop breathing		
No	5,678 (74.30%)	Ref
Yes	1,611 (21.08%)	3.06 (2.11, 4.00) <0.0001
Not recorded	353 (4.62%)	4.38 (2.54, 6.23) <0.0001
Overly sleepy		
No	1,365 (17.86%)	Ref
Yes	5,946 (77.81%)	−2.44 (−3.45, −1.43) <0.0001
Not recorded	8 (0.10%)	13.19 (−0.58, 26.97) 0.0606
Bedtime	4.88 ± 1.76	−0.56 (−0.78, −0.34) <0.0001

*Ref, reference; TPA, total physical activity; BMI, body mass index; GFR, glomerular filtration rate; UA, uric acid; HDL, high-density lipoprotein; TC, total cholesterol; LDL, low-density lipoprotein*.

### Association Between Bedtime at Night and SBP

**Figure 2:**
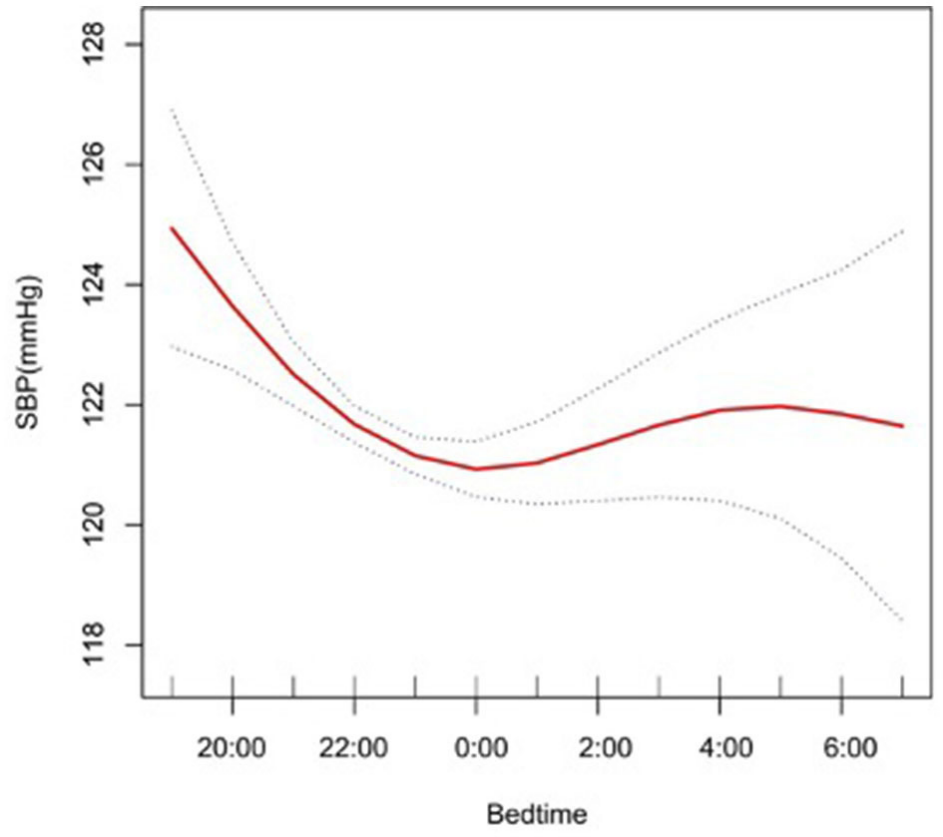
A smooth curve fitting for the relationship between bedtime at night and SBP. Adjust for gender, age, race, alcohol consumption, smoke, TPA, GFR, UA, BMI, diabetes, HDL, LDL, TC, sleep duration, snore, trouble sleeping, snort or stop breathing, overly sleepy.

The results from multiple linear regression analyses are illuminated in Table 4, which are used to explore the association between bedtime and SBP. In model I, after adjustment for sleep factors: sleep duration, snore, trouble sleeping, snort or stop breathing, and overly sleepy, a significantly negative association was identified (β, −0.74 [95% CI, −0.97, −0.51]). In model II, we adjusted for gender, race, and age in addition to the model I, a significantly negative association still presented (β, −0.26 [95% CI, −0.47, −0.05]). In model III, we adjusted for alcohol consumption, smoke, TPA, GFR, UA, TC, LDL, HDL, BMI, and diabetes in addition to model II, a significantly negative association still existed (β, −0.23 [95% CI, −0.43, −0.02]). We also conducted a smooth curve fitting to explore the non-linear relationship between bedtime and SBP (Figure 2). With the delay of bedtime, the SBP had a gradual decrease trend, which was decreased to the minimum at 0:00, and then increased gradually.

**Table 4:** Relationship between bedtime at night and SBP in different models.

**Exposure**	**Model I (β, 95%CI, P)**	**Model II (β, 95%CI, P)**	**Model III (β, 95%CI, P)**
Bedtime			
4.88 ± 1.76	−0.74 (−0.97, −0.51) <0.0001	−0.26 (−0.47, −0.05) 0.0155	−0.23 (−0.43, −0.02) 0.0292

*SBP, systolic blood pressure. Model I was adjusted for: sleep duration, snore, trouble sleeping, snort or stop breathing, overly sleepy. Model II was adjusted for: gender, race, age in addition to model I. Model III was adjusted for: alcohol consumption, smoke, TPA, GFR, UA, TC, LDL, HDL, BMI, diabetes in addition to model II*.

### Subgroup Analyses of Factors Influencing the Association Between Bedtime at Night and SBP

**Figure 3:**
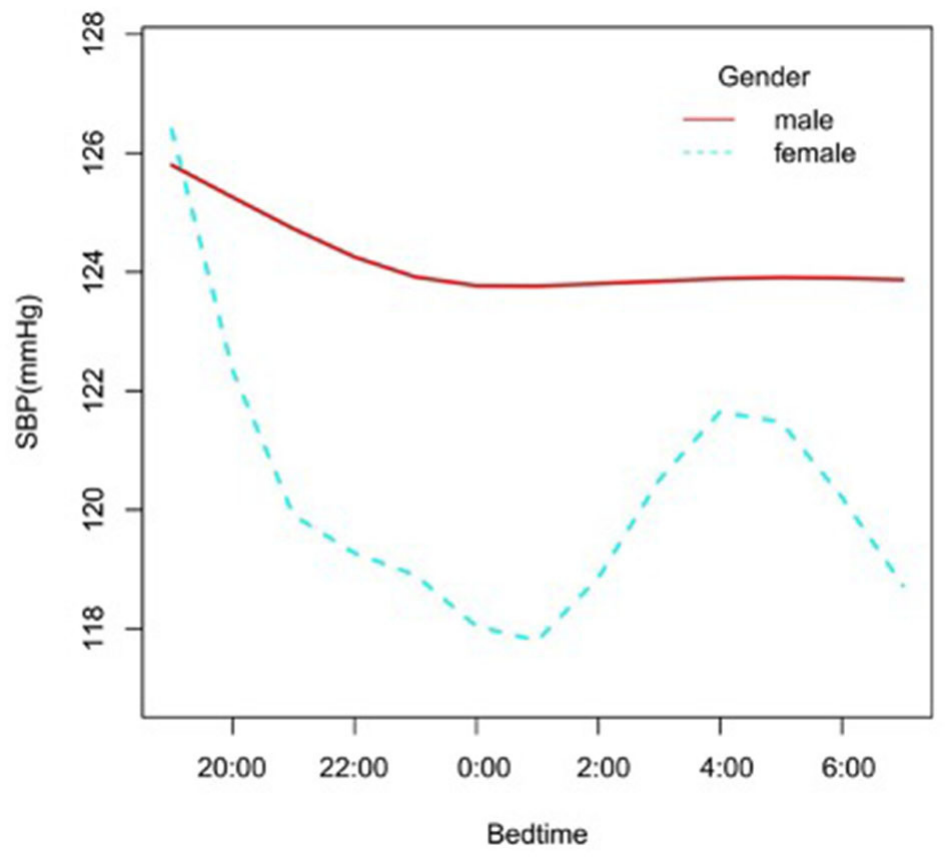
A smooth curve fitting for the relationship between bedtime at night and SBP based on different gender. Adjust for age, race, alcohol consumption, smoke, TPA, GFR, UA, BMI, diabetes, HDL, LDL, TC, sleep duration, snore, trouble sleeping, snort or stop breathing, overly sleepy.

**Figure 4:**
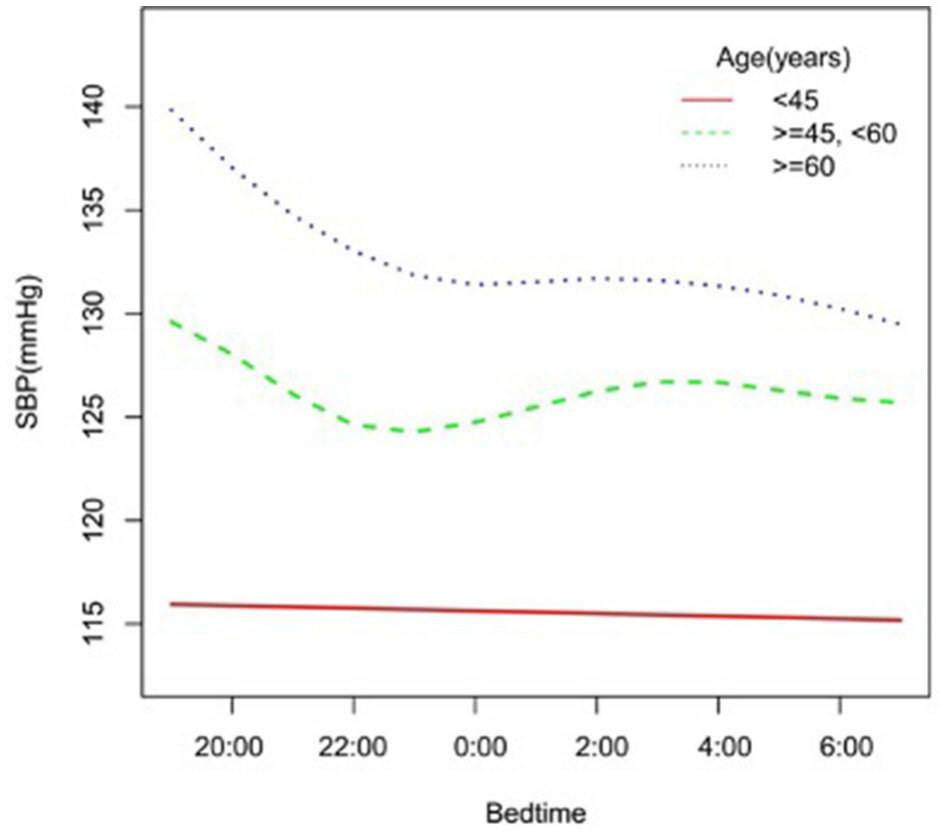
A smooth curve fitting for the relationship between bedtime at night and SBP based on different age. Adjust for gender, race, alcohol consumption, smoke, TPA, GFR, UA, BMI, diabetes, HDL, LDL, TC, sleep duration, snore, trouble sleeping, snort or stop breathing, overly sleepy.

**Figure 5:**
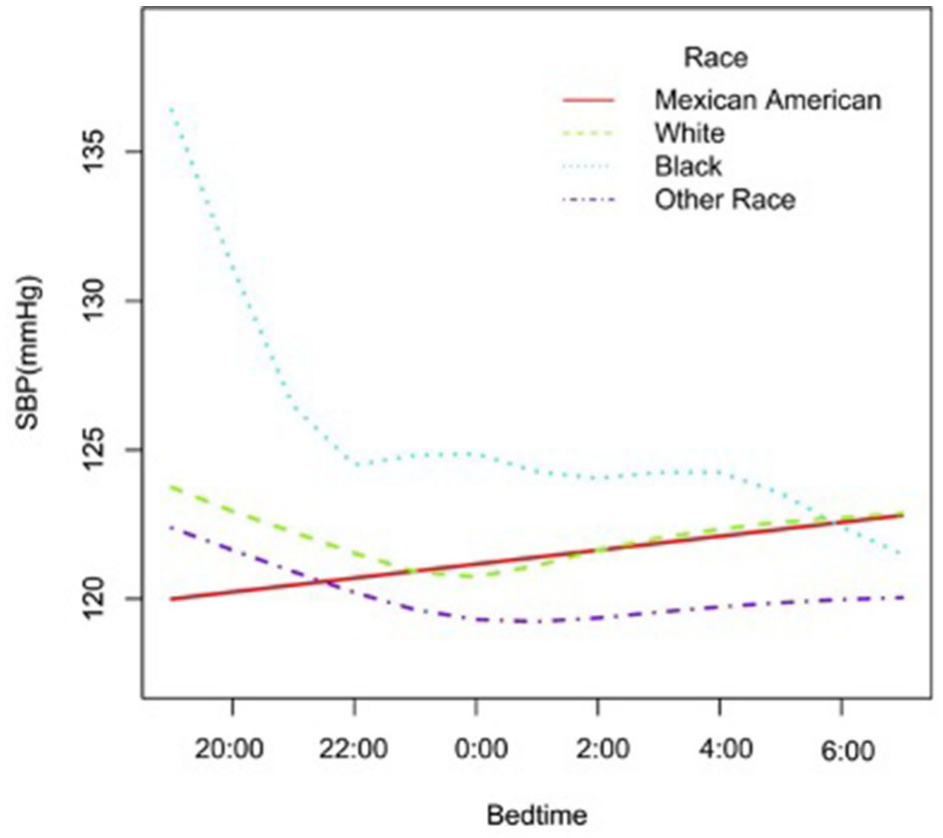
A smooth curve fitting for the relationship between bedtime at night and SBP based on different race. Adjust for gender, age, alcohol consumption, smoke, TPA, GFR, UA, BMI, diabetes, HDL, LDL, TC, sleep duration, snore, trouble sleeping, snort or stop breathing, overly sleepy.

**Figure 6:**
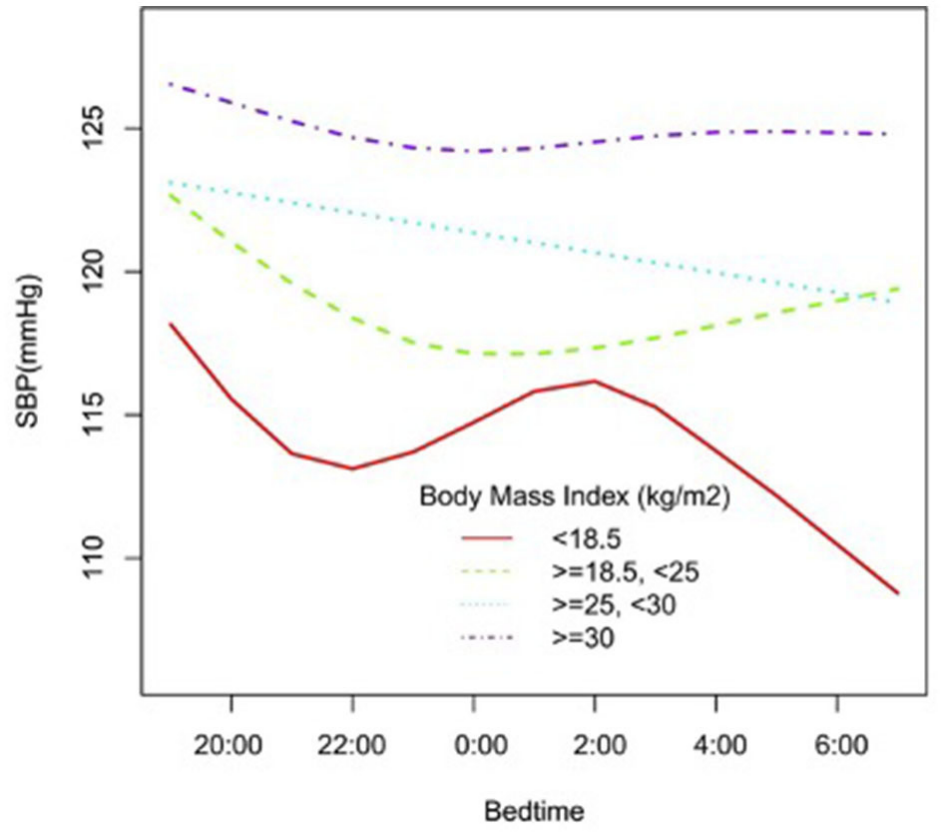
A smooth curve fitting for the relationship between bedtime at night and SBP based on different BMI. Adjust for gender, age, race, alcohol consumption, smoke, TPA, GFR, UA, diabetes, HDL, LDL, TC, sleep duration, snore, trouble sleeping, snort or stop breathing, overly sleepy.

In the subgroup analysis stratified by gender, race, age, and BMI, the association between bedtime and SBP is explored in Figures 2–6. All the potential confounding factors except the subgroup variable were adjusted. In the female group (Figure 3), with the delay of bedtime, the range of SBP was decreased more obviously at 0:00, then was increased gradually, and fell again after 4:00. For the second downward trend, we thought that the sample size at each time point was too small to reflect the true relationship. In different age groups (Figure 4), with the age increasing, BP was gradually increased. In the 18–44 year group, bedtime had little effect on SBP. Among the≥45 years old group, this trend also existed. In the Black group (Figure 5), an obvious downward trend was found at 22:00, and the SBP was the lowest. However, the trend was not found in Mexican Americans. In different BMI groups (Figure 6), the downward trend of SBP was more obvious in groups with 18.5–24.9 BMI. In the group with BMI ≤ 18.5, the sample size was too small to reflect the true relationship.

## Discussion

With the development of society and the economy, people's sleep patterns, including bedtime, wake time, and sleep duration, have been changed a lot. Sleep has been identified as a key lifestyle factor for cardiovascular disease (3). Many studies have explored the association between sleep duration and BP. A prospective cohort study, which included 162,121 adults, proved that self-reported short sleep duration (<6 h) increased the risk of hypertension (≥130/85 mmHg) by 8% {hazard ratio [HR] (95% CI) 1.08 (1.04–1.13)} (20). Another research with 71,455 participants demonstrated that compared with the 8 h group, people whose sleep duration was <6 h or 6 h were more likely to develop hypertension (odds ratio (OR):1.49 (1.34–1.64) and 1.15 (1.08–1.23), respectively) (8). As to long sleep duration, the association was not very clear. One study with 5,910 participants illuminated that compared to sleep duration with 7–8 h, the adjusted ORs of 8–9 and ≥9 of sleep duration to hypertension was 1.19 (1.04–1.37) and 1.30 (1.04–1.62), respectively (21). However, 2 meta-analyses concluded that long sleep duration was not related to the occurrence of hypertension (22, 23). Earlier bedtime or later bedtime generally means longer or shorter sleep duration. In our study, a U-shaped relationship was identified between bedtime and SBP. A prospective cohort study identified that among adolescents, compared to participants with weekday bedtime between 9 p.m. and 10 p.m., participants with bedtime at 11 p.m. or later on weekdays had a 1.87 times higher risk of increased BP during the follow-up period (95% CI = 1.09, 2.21). A participant with earlier bedtime on a weekday also had a higher risk of elevated BP (risk ratio = 1.96; 95% CI =1.27, 3.01) (24), which was similar compared with our research.

The biological mechanism of the relationship between sleep patterns and BP has not been clear yet. Circadian rhythms including body temperature, sleep-wake cycle, metabolism, and BP, were controlled by the central clock of the suprachiasmatic nucleus of the hypothalamus and the peripheral clocks throughout the body (25). BP was controlled by many different systems including the sympathetic nervous system, central nervous system, kidneys, heart, vascular system, and immune system, which ultimately lead to changes in cardiac output and peripheral resistance, thereby causing changes in BP (26). Different sleep duration or bedtime could disturb the circadian rhythm of BP, which would cause changes in autonomic nerve tension (7). Autonomic dysfunction, especially sympathetic activation, may lead to the occurrence and development of hypertension and the reduction of nocturnal dipping (27, 28). In epidemiological studies, short sleep duration and long sleep duration were both associated with increased incidence of obesity and type 2 diabetes (3), which also played a role in promoting the occurrence and development of hypertension. Falling asleep too early or too late would disrupt the normal sleep-wake cycle. Previous studies clarified that when a sleep-wake cycle of an individual was inconsistent with the external environment, the average arterial pressure would increase by 3% (29). Although the clinical effects of bedtime on BP may not be significant in all populations, these effects may be significant in some special populations. One meta-analysis evaluating the use of antihypertensive drugs to prevent cardiovascular disease showed that a decrease of 1 mmHg in SBP and a decrease of 0.5 mmHg in diastolic BP was associated with a 4% decrease in stroke and a 2% decrease in coronary heart disease events (30).

There were some limitations in our study. First, subjective bedtime may have measurement errors and recall biases, and the covariate self-reported sleep duration had only a moderate correlation with the objective sleep duration (31). Second, we did not consider other potential confounding factors, such as sleep quality, socio-economic status, and educational status. Further research studies need to be done to comprehensively explore the association of more factors with hypertension.

## Conclusions

With the delay of bedtime, the SBP showed a gradual decrease trend, and it dropped to the lowest at 0:00. After 0:00, the SBP was gradually increased with the delay of sleep time. A U-shaped relationship was identified between bedtime and SBP. In the female group, the range of SBP was decreased more obviously before 0:00.

## Data Availability Statement

Publicly available datasets were analyzed in this study. This data can be found at: https://www.cdc.gov/nchs/nhanes/index.htm.

## Author Contributions

YS and ND: conception, design, collection, and assembly of data. ND: administrative support. CL, YL, and LH: provision of study materials or patients. YS and LH: data analysis and interpretation. All authors final approval of manuscript.

## Conflict of Interest

The authors declare that the research was conducted in the absence of any commercial or financial relationships that could be construed as a potential conflict of interest.

## Publisher's Note

All claims expressed in this article are solely those of the authors and do not necessarily represent those of their affiliated organizations, or those of the publisher, the editors and the reviewers. Any product that may be evaluated in this article, or claim that may be made by its manufacturer, is not guaranteed or endorsed by the publisher.

## Abbreviations

UA: uric acid
SBP: systolic blood pressure
BP: blood pressure
BMI: body mass index
HDL: high-density lipoprotein
TC: total cholesterol
LDL: low-density lipoprotein
CI: confidence interval
OR: odds ratio
TPA: total physical activity
GFR: glomerular filtration rate.
